# Supplementary material for: Foxc1 establishes enhancer accessibility for craniofacial cartilage differentiation
Source: eLife. 2021 Jan 27;10:e63595. doi: 10.7554/eLife.63595 (PMC7891931; doi:10.7554/eLife.63595)
Supplement: Supplementary file 1. — Detailed lists of predicted motifs for cartilage-accessible elements in 72 hpf controls (A), and based on timing of gain of accessibility (B), and dependence on Foxc1 function (C). [file elife-63595-supp1.docx]

**Supplementary file 1A. Motifs recovered from elements with selective accessibility in *sox10:Dsred*+; *col2a1a*:GFP+ chondrocytes at 72 hpf.**

| Top 500 | | | Top 1000 | | | Top 2000 | | |
| --- | --- | --- | --- | --- | --- | --- | --- | --- |
| TF motifs | P value | % of targets | TF motifs | P value | % of targets | TF motifs | P value | % of targets |
| Sox | 1e-54 | 50.40 | Sox | 1e-105 | 45.60 | Sox | 1e-206 | 29.35 |
| Fox | 1e-31 | 36.00 | Tead | 1e-49 | 57.90 | Fox | 1e-107 | 36.75 |
| Tead | 1e-28 | 39.60 | Nfat | 1e-30 | 30.30 | Nfat | 1e-88 | 31.60 |
| Nfat | 1e-20 | 1.6 | Egr | 1e-65 | 55.47 | Zfx | 1e-71 | 47.80 |
| Smad | 1e-18 | 37.40 | Fox | 1e-28 | 35.10 | Nkx | 1e-53 | 20.05 |
| Tcf/Lef | 1e-18 | 26.80 | Nr | 1e-25 | 36.30 | Nr | 1e-49 | 70.55 |
| Nr | 1e-17 | 1.40 | Nkx | 1e-21 | 34.80 | Irf | 1e-38 | 62.40 |
| Klf | 1e-17 | 34.40 | Osr | 1e-21 | 9.70 | Klf | 1e-37 | 34.00 |
| Plag | 1e-15 | 1.50 | Ap1 | 1e-20 | 20.30 | Myf | 1e-33 | 23.35 |
| Ets | 1e-15 | 34.20 | Hox | 1e-20 | 7.50 | Mef2 | 1e-32 | 5.95 |
| Hox | 1e-15 | 2.40 | Ets | 1e-19 | 54.30 | Ap1 | 1e-31 | 11.45 |
| Zfx | 1e-15 | 2.80 | Klf | 1e-19 | 48.40 | Atf | 1e-30 | 12.10 |
| Cdx | 1e-14 | 1.20 | Creb | 1e-19 | 22.50 | Sp1 | 1e-29 | 9.30 |
| Nkx | 1e-14 | 1.20 | Plag | 1e-18 | 25.89 | Tead | 1e-29 | 8.80 |
| Gfi1b | 1e-14 | 3.60 | Runx | 1e-17 | 0.80 | Tcf/Lef | 1e-26 | 22.95 |
| Pax | 1e-14 | 1.40 | Hbp | 1e-16 | 4.00 | Ets | 1e-21 | 9.15 |
| Spib | 1e-13 | 2.00 | Meis | 1e-15 | 10.30 | Runx | 1e-20 | 19.15 |
| Irf | 1e-12 | 1.20 | Egr | 1e-14 | 1.40 | Rxra | 1e-20 | 0.85 |
| E2f6 | 1e-12 | 8.00 | Mef2 | 1e-13 | 12.10 | Smad | 1e-19 | 9.15 |
| Runx | 1e-12 | 1.60 | Zfx | 1e-13 | 0.70 | Mitf | 1e-17 | 0.45 |

**Supplementary file 1B. Motifs recovered from the three groups of cartilage-accessible elements based on their timing of gain of accessibility.**

| Group I | | | Group II | | | Group III | | |
| --- | --- | --- | --- | --- | --- | --- | --- | --- |
| TF motifs | P value | % of targets | TF motifs | P value | % of targets | TF motifs | P value | % of targets |
| Nfat | 1e-24 | 20.06 | Sox | 1e-445 | 36.54 | Nfat | 1e-155 | 33.18 |
| Foxa2 | 1e-23 | 60.17 | Foxa1 | 1e-106 | 36.80 | Foxa1 | 1e-110 | 47.20 |
| Lhx | 1e-21 | 12.15 | Nfat | 1e-91 | 45.93 | Sox | 1e-73 | 37.61 |
| Nr2f | 1e-18 | 48.59 | Egr | 1e-65 | 55.47 | Ap1 | 1e-72 | 11.67 |
| Meis | 1e-17 | 15.54 | Ap1 | 1e-60 | 13.42 | Tead | 1e-63 | 36.17 |
| Pax | 1e-17 | 3.39 | Nkx***** | 1e-58 | 35.80 | Irf****** | 1e-54 | 29.39 |
| Tead | 1e-17 | 8.47 | Ets | 1e-53 | 65.50 | Dlx****** | 1e-51 | 23.98 |
| Ets | 1e-17 | 23.45 | Mafk***** | 1e-44 | 21.56 | Ets | 1e-51 | 12.69 |
| Nkx | 1e-17 | 6.21 | Tcf | 1e-42 | 6.99 | Runx | 1e-46 | 18.79 |
| Hox | 1e-16 | 10.45 | Fra1***** | 1e-31 | 28.48 | Myf | 1e-44 | 30.19 |
| E2f | 1e-16 | 2.26 | Mef2***** | 1e-27 | 4.11 | Atf | 1e-37 | 26.44 |
| Sox | 1e-15 | 1.69 | Spib***** | 1e-25 | 8.99 | Tcf | 1e-37 | 25.64 |
| Nr | 1e-15 | 1.69 | Myf | 1e-22 | 1.55 | Myb | 1e-36 | 17.27 |
| Rxra | 1e-15 | 1.69 | Atf | 1e-21 | 25.89 | E2f****** | 1e-36 | 25.15 |
| Prdm | 1e-15 | 48.31 | Tead | 1e-20 | 3.33 | Tbx****** | 1e-30 | 13.48 |
| Irf | 1e-15 | 5.93 | Zf***** | 1e-18 | 0.37 | Hox | 1e-23 | 4.47 |
| Runx | 1e-15 | 3.39 | Hox | 1e-17 | 0.41 | Meis****** | 1e-22 | 21.44 |
| Tcf | 1e-14 | 24.29 | Mtf***** | 1e-16 | 0.48 | Smad4****** | 1e-21 | 21.33 |
| Ap1 | 1e-13 | 2.26 | Runx | 1e-16 | 4.62 | Klf****** | 1e-21 | 9.96 |
| Cdx | 1e-12 | 6.21 | Myb | 1e-16 | 7.58 | Egr | 1e-20 | 15.53 |

* Unique TFs recovered from Group II

** Unique TFs recovered from Group III

**Supplementary file 1C. Motifs recovered from Foxc1-dependent and -independent cartilage-accessible elements.**

| Group I | | | | | | Group II | | | | | |
| --- | --- | --- | --- | --- | --- | --- | --- | --- | --- | --- | --- |
| Foxc1-dependent* | | | Foxc1-independent | | | Foxc1-dependent | | | Foxc1-independent | | |
| TF motifs | P value | % of targets | TF motifs | P value | % of targets | TF motifs | P value | % of targets | TF motifs | P value | % of targets |
| Foxa2 | 1e-45 | 37.50 | Sox | 1e-76 | 37.87 | Sox | 1e-113 | 37.26 | Sox | 1e-188 | 32.39 |
| Foxd3 | 1e-16 | 18.33 | Ets | 1e-61 | 20.53 | Foxa1 | 1e-59 | 38.05 | Myf | 1e-51 | 70.98 |
| Meis | 1e-15 | 8.33 | Foxo1 | 1e-42 | 48.50 | Sox | 1e-43 | 32.08 | Nfat | 1e-41 | 15.54 |
| Tead | 1e-14 | 34.17 | Insm | 1e-39 | 29.79 | Zfx | 1e-24 | 26.10 | Nkx | 1e-35 | 52.39 |
| Mtf | 1e-12 | 4.17 | Ap1 | 1e-35 | 13.90 | Nfat | 1e-23 | 11.32 | Tcf/lef | 1e-33 | 13.37 |
| Sox | 1e-12 | 4.17 | Nfat | 1e-33 | 38.24 | Runx | 1e-23 | 36.95 | Zfx | 1e-27 | 6.85 |
| Ets | 1e-12 | 11.67 | Klf | 1e-24 | 43.42 | Nr | 1e-21 | 24.69 | Ets | 1e-24 | 20.43 |
|  |  |  | Hox | 1e-23 | 8.90 | Stat | 1e-18 | 4.09 | Foxh1 | 1e-22 | 21.63 |
|  |  |  | Mtf | 1e-22 | 0.91 | Tead | 1e-17 | 10.85 | Myc | 1e-21 | 4.78 |
|  |  |  | Zf | 1e-22 | 28.79 | Mef2 | 1e-16 | 23.74 | Zf | 1e-19 | 3.70 |
|  |  |  | Gfi | 1e-20 | 28.97 | Ap1 | 1e-16 | 1.10 | Myf | 1e-44 | 30.19 |
|  |  |  | Tead | 1e-20 | 6.18 | Maf | 1e-15 | 4.09 | Foxp1 | 1e-19 | 6.52 |
|  |  |  | Spib | 1e-19 | 0.91 | Tbx | 1e-14 | 7.08 | Tcf | 1e-18 | 0.87 |
|  |  |  | Tcf | 1e-19 | 1.91 | Irf | 1e-14 | 0.94 | Irx | 1e-17 | 1.20 |
|  |  |  | Mef2 | 1e-18 | 5.36 | Ets | 1e-14 | 13.21 | Nr | 1e-16 | 15.33 |
|  |  |  | Rxra | 1e-18 | 5.45 | Tcf | 1e-14 | 1.26 | E2f | 1e-16 | 3.37 |
|  |  |  | Ap1 | 1e-17 | 0.73 | Smad2 | 1e-13 | 7.39 | Irf | 1e-15 | 4.13 |
|  |  |  | E2f6 | 1e-16 | 7.27 | Hox | 1e-13 | 1.26 | Egr | 1e-15 | 0.76 |
|  |  |  | Tfap | 1e-16 | 0.91 | Mafk | 1e-12 | 2.99 | Nanog | 1e-14 | 3.48 |
|  |  |  | Smad2 | 1e-15 | 0.82 | Mtf | 1e-12 | 0.94 | Prdm | 1e-14 | 12.07 |

* Only TFs with p value < 1e-11 are shown.
