# Supplementary material for: Foxc1 establishes enhancer accessibility for craniofacial cartilage differentiation
Source: eLife. 2021 Jan 27;10:e63595. doi: 10.7554/eLife.63595 (PMC7891931; doi:10.7554/eLife.63595)
Supplement: Supplementary file 2. — Description of independent alleles for each enhancer transgenic line, genomic coordinates of elements tested, and activity in cartilage in 6 dpf zebrafish. [file elife-63595-supp2.docx]

**Supplementary file 2. Summary of transgenic analysis of cartilage-accessible elements.**

| Accessible element | Coordinates (GRCz10) | Cartilage activity | Accessible element | Coordinates (GRCz10) | Cartilage activity |
| --- | --- | --- | --- | --- | --- |
| *sox10_p1*^;^*** | chr3:2,012,803-2,013,385 | N | *gas1b_p1* | chr5:31,759,240-31,759,695 | Y (Fig 4-S1) |
| *sox10_p2*  *(el810, el815, el816)* | chr3:2,014,559-2,015,255 | Y (Fig 4) | *gas1b_p2***  *(el844)* | chr5:31,765,440-31,766,403 | Y (Fig 4) |
| *ucmab_p1*  *(el806, el807)* | chr25:7,856,000-7,856,500 | Y (Fig 4) | *acana_p1*  *(el831, el832)* | chr7:48,479,150-48,479,550 | Y (Fig 4-S1) |
| *lect1_p1*  *(el826)* | chr9:54,191,100-54,191,550 | Y (Fig 4) | *acana_p2* | chr7:48,481,700-48,482,152 | N |
| *lect1_p2* | chr9:54,169,800-54,170,250 | N | *acana_p3* | chr7:48,487,926-48,488,318 | Y |
| *lect1_p3**  *(el813, el814, el821)* | chr9:54,185,300-54,185,701 | Y (Fig 4) | *slc35d1a_p1*  *(el850)* | chr6:34,871,038-34,872,042 | Y (Fig 4-S1) |
| *col9a3_p1*  *(el817, el830)* | chr23:510,170-510,510 | Y (Fig 4) | *sparc_p1***  *(el843)* | chr14:25,662,146-25,662,540 | Y (Fig 4-S1) |
| *epyc_p1*  *(el811, el812)* | chr4:16,343,350-16344000 | Y (Fig 4) | *foxa3_p1* | chr18:46,356,160-46,356,470 | N |
| *epyc_p2* | chr4:16,344,600-16,345,300 | N | *si:dkey33i11.4_p1* | chr16:45,266,645-45,267,035 | N |
| *mia_p1*  *(el822, el823)* | chr5:56,986,683-56,987,239 | Y (Fig 4) | *lefty2_p1* | chr17:8,170,102-8,170,632 | N |
| *matn1_p1* | chr19:43,722,488-43,723,132 | N | *emx3_p1**;#*  *(el853)* | chr14: 26,076,432-26,076,922 | N (Fig 4-S2) |
| *matn1_p2** | chr19:43,718,561-43,719,175 | N | *satb2_p1**;#* | chr9:32,768,758- 32,769,253 | N (Fig 4-S2) |
| *col9a1a_p1*  *(el824, el825)* | chr11:11,303,150-11,303,700 | Y (Fig 4-S1) | *prrx1a_p1**;#*  *(el852)* | chr2:23,506,193- 23,506,666 | N (Fig 4-S2) |
| *matn4_p1* | chr6:52,777,100-52,777,600 | N | *prrx1a_p2**;#* | chr2:23,518,281- 23,518,832 | N |
| *matn4_p2**  *(el846, el847)* | chr6:52,802,061-52,802,624 | Y (Fig 4) | *cd248a_p1**;#* | chr7:18,098,986- 18,099,39 | N |
| *matn4_p3*** | chr6:52,794,357-52,794,959 | Y (Fig 4-S1) |  |  |  |

* Indicate peaks locate in the promoter regions.

** Indicate Foxc1-independent peaks.

# Indicate Group I peaks.

Independent founder alleles are listed below the accessible elements for which stable lines were analyzed. All other elements were assayed in injected embryos.
