# Supplementary material for: Foxc1 establishes enhancer accessibility for craniofacial cartilage differentiation
Source: eLife. 2021 Jan 27;10:e63595. doi: 10.7554/eLife.63595 (PMC7891931; doi:10.7554/eLife.63595)
Supplement: Supplementary file 3. — Details on primer sequences used to amplify probe regions for cloning, enyzmes used to linearize the probe plasmids, and the types of RNA polymerase used to synthesize RNA probes for in situ hybridization. [file elife-63595-supp3.docx]

**Supplementary file 3. In situ probes**

| **Gene** | **Forward Primer (5’-3’)** | **Reverse Primer (5’-3’)** | **Enzyme for linearization** | **RNA**  **Polymerase** |
| --- | --- | --- | --- | --- |
| *sox10* | GGAACTGCAGGGAGGAAAAT | AGGCGAGTGTTTCGATGATT | BamHI | T7 |
| *col9a3* | GGTCCAACTGGAATCAGAGG | TAATGGTTCTTGGGGTTGGA | EcoRV | SP6 |
| *lect1* | CCACTGAAGGACAGCAGCTT | AAGGAAATGCATGGCGATTA | EcoRV | Sp6 |
| *epyc* | GACCTCAAGTTGGACCGTGT | CACAAGCAGCGTAGAGTTGG | BamHI | T7 |
